# The perception of time is slowed in response to exercise, an effect not further compounded by competitors: behavioral implications for exercise and health

**DOI:** 10.1002/brb3.3471

**Published:** 2024-04-01

**Authors:** Andrew Mark Edwards, Stein Gerrit Paul Menting, Marije Titia Elferink‐Gemser, Florentina Johanna Hettinga

**Affiliations:** ^1^ School of Psychology and Life Sciences Canterbury Christ Church University Canterbury UK; ^2^ Center for Human Movement Sciences University Medical Center Groningen, University of Groningen Groningen The Netherlands; ^3^ Department of Sport, Exercise & Rehabilitation, Faculty of Health and Life Sciences Northumbria University Newcastle UK

**Keywords:** competition, exercise‐behavior, pacing, self‐regulation, time perception

## Abstract

**Introduction:**

The theory of relativity postulates that time is relative to context and exercise seems such a situation. The purpose of this study was to examine whether situational factors such as perceived exertion and the introduction of an opponent influence competitors’ perception of time.

**Methods:**

Thirty‐three recreationally active adults (F = 16; M = 17) performed three standardized 4‐km cycling trials in a randomized order. Velotron 3D software was used to create a visual, virtual environment representing (1) a solo time trial (FAM and SO), (2) a time trial with a passive opponent avatar (PO), and (3) a time trial with an opponent avatar and participant instruction to actively finish the trial before the opponent (AO). Participants were asked to estimate a 30‐s time period using a standardized protocol for reproducibility before exercise at 500 m, 1500 m, 2500 m, and post exercise. Rate of perceived exertion (RPE) was measured throughout the trials.

**Results:**

Exercise trials revealed that time was perceived to run “slow” compared to chronological time during exercise compared to resting and post‐exercise measurements (*p* < 0.001). There was no difference between exercise conditions (SO, PO, and AO) or time points (500 m, 1500 m, and 2500 m). RPE increased throughout the trials.

**Conclusion:**

The results of this study demonstrate for the first time that exercise both with and without the influence of opponents influences time perception. This finding has important implications for healthy exercise choices and also for optimal performance. Independent of RPE, time was perceived to move slower during exercise, underpinning inaccurate pacing and decision‐making across physical activities.

## Graphical abstract

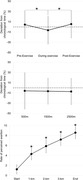

## 1 INTRODUCTION

Einstein's theory of relativity recognized the similarity between the relativity of physical and psychological time over a century ago: “When a man sits with a pretty girl for an hour, it seems like a minute. But let him sit on a hot stove for a minute—and it's longer than any hour. That's relativity” Buhusi & Meck, 2009. The relativity of psychological time is still debated and this has not been extensively investigated in the psychophysiological literature related to the impacts of time perception in sport and exercise. Previous studies (Edwards & McCormick, 2017; Hanson & Lee, 2020) have demonstrated that exercise distorts the perception of time (subjective time), giving the sense that time has slowed down, resulting in an under‐estimation of (chronological) time elapsed. It has been suggested that this observation could be impactful for competitive athletes racing against the clock and/or opponents (Edwards & McCormick, 2017). However, there remains minimal research in this area (Behm & Carter, 2020; Edwards & McCormick, 2017; Hanson & Buckworth, 2016; Hanson & Lee, 2020). Thus far, the perceived slowing of elapsed time during exercise has only been shown in capped exercise at fixed intensities and has not yet been shown during self‐paced exercise akin to that of competitive sport. This is surprising given how important accurate pacing is to athletic performance in competitive sport (Edwards & Polman, 2012). Whether time is perceived to run fast or slow, distortion from chronologic time therefore likely impedes performance.

Many diverse processes are involved in psychological timing and it is now clear that numerous brain areas are involved in the experiencing of time (Block & Gruber, 2014). The study of time perception, therefore, poses unique challenges as there is no single mechanistic pathway or time‐sense organ carrying temporal information from the periphery to the brain. These factors complicate the search for an overarching, explanatory information processing model. Fundamentally, there currently are two predominant theories, the scalar expectancy theory (SET) and the striatal beat frequency (SBT) model, which co‐exist and are neatly summarized elsewhere (Behm & Carter, 2020). These two theories emphasize the perception of the number of events (impulses) in a period and the role of neurotransmitters in activating and coordinating cortical structures, respectively, to influence the attentional processes and capacity of the brain. Time perception in response to exercise appears to draw on elements of both theories insofar as SET posits the influence of experience and task‐specific memory common to accurate pacing (Menting et al., 2022), while the environmental conditions and physical sensations of exercise (Smits et al., 2014) are likely to influence the perception of impulse frequency integral to the striated beat frequency model. Indeed, evidence suggests that when greater attentional resources are placed on a temporal judgment task it tends to subjectively lengthen perceived time (Block & Gruber, 2014). However, it is increasingly clear that the judgment of a given duration depends on both the non‐temporal properties of the stimuli that define the time interval (e.g. the environment) and the task methodology used to elicit a duration judgment. In other words, the subjective perception of time can be distorted in either direction such as appearing to run quickly when diverting attention away from a focus on time elapsed, or running slowly when increasing the attentional focus on to time such as through experiencing pain sensations, leading to a more associative (i.e., in the moment) compared to a dissociative (i.e., distracted from the moment) state.

Applying this in a sporting context, as the exercise‐induced negative pain signals reach the brain, increasing attention, individuals reach a highly associative state of impulse awareness and focus more on the time elapsed, resulting in a perceived slowing of time (Edwards & Polman, 2013). In the limited studies investigating time perception in response to exercise, it has been confirmed that time appears to run more slowly at higher intensities but as yet, this has not been fully quantified in relation to resting conditions (Hanson & Lee, 2020).

In clinical situations, it has previously been shown that individuals experiencing significant pain, such as during chemotherapy treatment, can to some extent be distracted from pain sensations by focusing attention on virtual reality software images. This appears to give patients the impression of time running faster than usual, resulting in an overestimation of time due to distraction and a more enjoyable experience (Gable & Poole, 2012; Schneider et al., 2011). External stimuli such as the presence of opponents, peers, or the crowd could similarly divert attention, creating a moderately dissociative state for athletes that lessens their cognitive awareness of physical discomfort and distorts time in the opposite direct to exercise itself. Therefore, while exercise alone slows the perception of time, opponents could lessen the extent to which time is slowed (or perhaps could speed up) during exercise, creating a pseudo‐equilibrium. The practical potential of external stimuli to impact time perception during exercise has been demonstrated neatly through the use of a new LED light technology in track running (Wavelight Technology). This is a visual pace‐setting system utilizing a series of moving, illuminated LED lights on the inside of an athletics track, helping athletes to keep track with a target pace. Using this technology, several long‐standing track running world records have recently been broken, such as the men's 10,000 m (15‐year‐old record; broken in 2020) and the women's 5000 m (12‐year‐old record; broken in 2020). Studying the presence and/or impact of opponents or other external stimuli on the perception of time during exercise is of importance to properly understand and strategize how to use this information constructively for performance enhancement. Opponents are known to be important determinants for an athlete's pacing decisions and, depending on the goal of the exercise, impact the effort expenditure in the initial phase of the trial (Konings & Hettinga, 2018) and the end‐spurt (do Carmo et al., 2022). However, as yet, the impact of external stimuli such as opponents in a competitive sport situation have not been investigated in relation to time perception.

The purpose of this study is first to confirm and extend earlier observations (Edwards & McCormick, 2017; Hanson & Lee, 2020) that the associative state caused by exercise results in individuals perceiving time to slow down, and measure this observation using standardized tests for the first time in uncapped, maximal self‐paced exercise trials resembling competitive sport. In addition, this study further aims to investigate whether various external stimuli, such as the presence of opponents as well as the level of engagement with this opponent, might provide a distraction that could counteract this perceived slowing of time during exercise or compound the problem. These combined goals may further elucidate the role of time perception in accurately judging pace in competitive sports situations with consequent impacts to training and performance.

## 2 METHODS

### 2.1 Participants

A total of 33 participants (16 female, 25.9 ± 3.3 years old, 172.3 ± 9.0 cm, 71.1 ± 12.5 kg) were recruited to participate in the study. The participants were moderate (*n* = 6) or highly active (*n* = 27) as assessed by the short version of the IPAQ (Dinger et al., 2006). Yet, none of the participants reported cycling as their sport of choice. The participants did not have previous experience in performing a cycling time trial. All participants were healthy and able to safely engage in physical activity, as assessed by the general health questions of the 2018 version of the PAR‐Q+ (Shephard et al., 1991; Warburton et al., 2018). Written informed consent was obtained from the participants during the first visit to the laboratory. The study was approved by the ethical committee of the local university (Northumbria University) in accordance with the Declaration of Helsinki. The authors are committed to open science.

### 2.2 Experimental procedure

#### 2.2.1 Time perception task

To standardize the approach to measuring time perception, a testing technique was developed to ensure subsequent reproducibility of the experimental protocol. Therefore, commencement was verbalized by indication of a start point “start” and participants were asked to estimate perceived time duration by verbally expressing “end” when they believed a 30‐ or 60‐s period of time had elapsed. Using a stopwatch, chronological time was recorded. Participants were not told what their estimated time was, as feedback could have affected performance on the following task. Participants subsequently performed the 30‐s time perception task before the 4‐km cycling trial, during the trial at either 500 m, 1500 m or 2500 m and 2 min after finishing the trial. The rate of perceived exertion (RPE) was asked pre‐ and post‐exercise as well as during exercise at 1, 2 or 3 km using the OMNI 0–10 cycling scale (Robertson et al., 2004). Measurement moments for time perception and RPE were randomized over the visits and between participants (Table 1).

**TABLE 1:** Overview of the randomized options for measurement points of time perception and rate of perceived exertion (RPE) during the 4‐km cycling trial.

	Option 1	Option 2	Option 3
Time perception	500 m	1500 m	2500 m
RPE moment 1	2 km	1 km	1 km
RPE moment 2	3 km	3 km	2 km

#### 2.2.2 Exercise trial

All cycling was performed on the Velotron cycling ergometer (Velotron Dynafit, Racermate). Using the Velotron 3D software, a 4‐km straight course was created and projected on a large screen in front of the participant. Competitors were visually represented by an on‐screen avatar. Before the exercise trial, the participants performed a 7‐min submaximal cycling trial as warming up. The first visit was used as a familiarization trial (FAM), in which the participants performed the 4‐km cycling trial with only their own avatar visible, and while receiving the instruction: “try to finish the 4‐km cycling trial as fast as possible”. During visits two, three and four, the participants performed the trial in three different conditions: (1) solo where only the participants’ avatar was visible and the goal was to try to complete the trial as fast as possible (SO), (2) alongside the participants’ avatar, another passive companion avatar was visible and the goal was to try to complete the trial as fast as possible (PO), or (3) alongside the participants’ avatar, an opponent avatar was visible and the goal was to try to competitively complete the trial before the active opponent (AO). These conditions were chosen as they represent an increasing presence and dependency upon the opponent avatar, which has previously been demonstrated to increase gaze fixation and associated attention (Konings et al., 2020). The order in which the conditions were performed was randomized. Each participant number had a predetermined order of conditions, which repeated itself after every six participants. The finish time of the other avatars was pre‐determined to be 105% of the participants’ finish time during FAM, which was chosen to offset the performance improvement in participants unfamiliar with a cycling trial as well as provide a stimulus to drive performance engagement (Konings et al., 2016; Menting et al., 2019). Participants were told the opponent was of a similar performance level as the participants. To replicate a real‐life race situation, and minimize influence on the time perception task, participants received no numerical feedback on heartrate, power output, velocity, time passed, the distance covered or left. Trials were conducted in ambient temperature between 19°C and 21°C.

### 2.3 Data analysis

A repeated measures analysis of variance (ANOVA) was performed to test the effect of exercise and the presence of an opponent on perceived time, using the measurement points (pre‐exercise, during exercise, and post‐exercise) and conditions (SO, PO, AO) as main effect within‐subject factors, as well as investigating the interaction. Post hoc analysis, using paired sample *t*‐tests with Bonferroni correction, was used to differentiate between time points and conditions. A two‐way ANOVA was used to investigate the difference in time perception throughout the trial (500, 1000, and 1500 m) and during the different conditions (SO, PO, and AO). A repeated measures ANOVA was used to test the effect of the differing conditions during exercise (SO, PO, and AO) on finish time of the 4‐km trial. A post hoc paired sample *t*‐test with Bonferroni correction was used to differentiate between conditions. Additionally, a two‐way ANOVA was used to analyze the differences in RPE throughout the trial (start, 1 km, 2 km, 3 km, and finish) and between conditions (SO, PO, and AO). For all ANOVAs, sphericity was tested using Mauchly's test of sphericity. The Greenhouse–Geisser correction was used as correction when the assumption was violated. Effect size of the test of variance was reported using Cohens’ *f*, and for all paired sample *t*‐tests reported using Cohen's *d*, indicating either a small (*f* ≤ 0.1 or *d* ≤ 0.2), medium (0.1 < *f* ≤ 0.4 or 0.2 < *d* ≤ 0.5), or large effect (*f* ≥ 0.4 or *d* ≥ 0.8).

## 3 RESULTS

Baseline pre‐exercise testing of the passive time perception assessment tasks did not reveal a difference between 30‐ and 60‐s estimates and therefore 30‐s samples were subsequently used in exercise trials for convenience.

**FIGURE 1:**
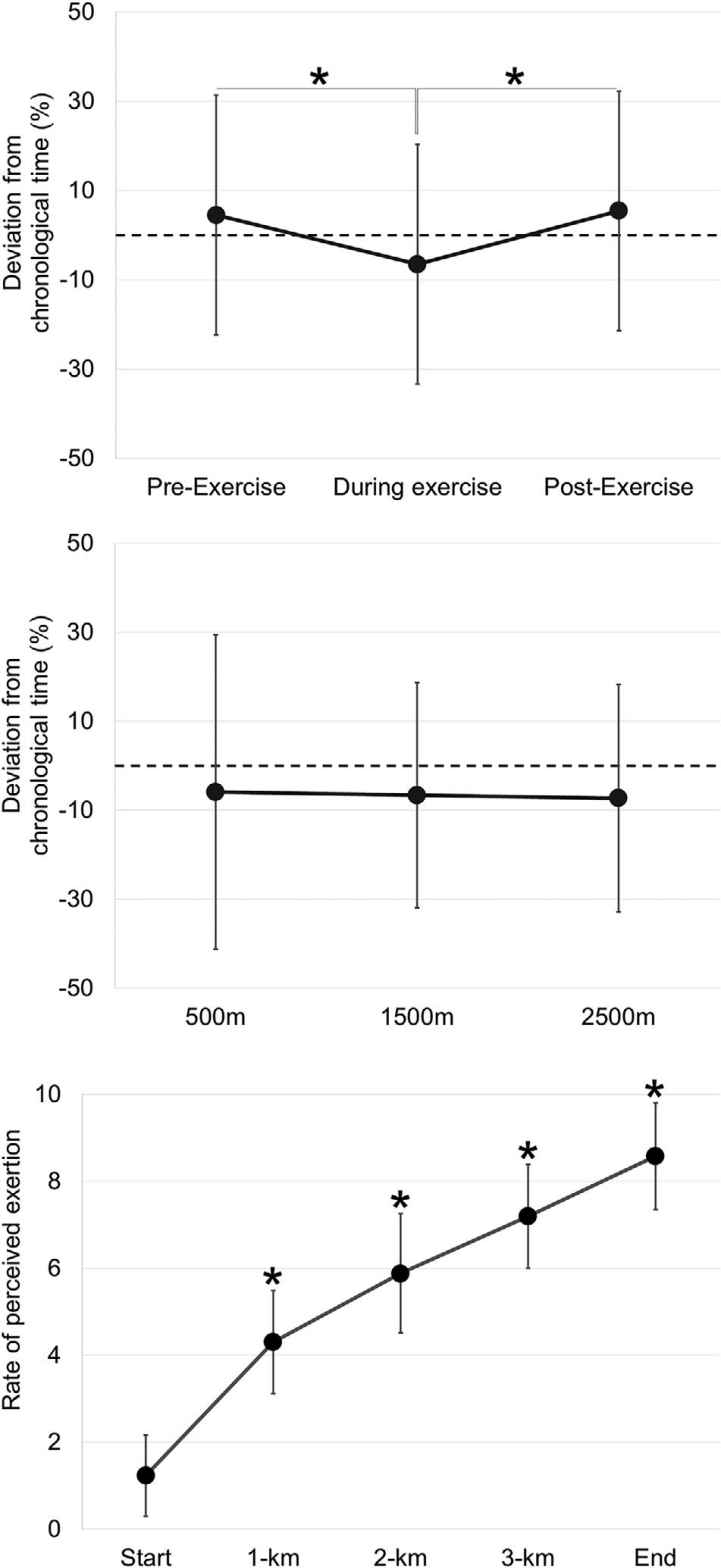
Mean (± SD) percentage difference between chronological time and perceived time pre‐exercise, during, and post‐exercise (a), as well as at 500, 1500, and 2500 m (b). Dotted line represents perceived time (30 s). The rate of perceived exertion at the start, 1 km, 2 km, 3 km, and the finish (c). *p < 0.01, *d* > 0.50 (in case of RPE, significantly different from previous).

The mean (± SD) chronological time measured pre‐exercise, during, and post‐exercise, and the RPE within the 4‐km trials are presented in Figure 1. The main effect of measurement point was significant (*F*_1.52, 48.56_ = 7.88, *p* < 0.01, *f* = 0.50). Post hoc testing demonstrated that time was perceived to move slower during the 4‐km exercise trials, compared to pre‐exercise and post‐exercise (*p* < 0.01, *d* = 0.50 and *p* < 0.01, *d* = 0.67, respectively). There is no difference between pre‐exercise and post‐exercise (*p* = 0.34, *d* = 0.07) (Figure 1a). The main effect of within‐trial measurement moment was not significant, indicating there was no difference in time perception between the different within‐trial measurement moments (500, 1500, and 2500 m) (*F*_4,90 _= 0.06, *p* = 0.95, *f* = 0.03) (Figure 1b). The significant main effect for measurement point demonstrated that RPE differed through the exercise trial (*F*_4,380_ = 582.39, *p* < 0.001, *f* = 2.48), being significantly higher at each measurement point (Figure 1c).

**FIGURE 2:**
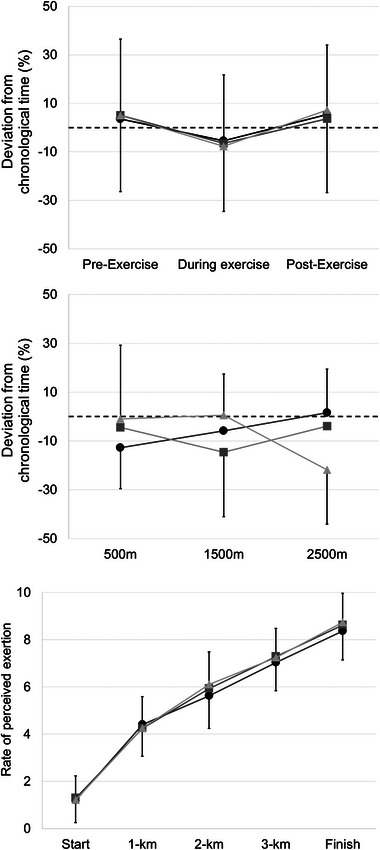
Mean (± SD) percentage difference between chronological time and perceived time pre‐exercise, during, and post‐exercise (a), as well as at 500, 1500, and 2500 m (b) within the different exercise conditions (SO is black circles, PO is dark gray squares, and AO is light gray triangles). Dotted line represents perceived time (30 s). The rate of perceived exertion at the start, 1 km, 2 km, 3 km, and the finish (c).

Comparing the finish times between conditions revealed a significant effect (*F*_2, 64 _= 3.88, *p* < 0.05, *f* = 0.35). The participants finished the 4‐km trial in the shortest time in the exercise trial with a contemporary on‐screen avatar where the instruction was to win (AO: 459.28 ± 37.92 s), compared to the trial with the presence of a contemporary avatar but the instruction to finish the trial as fast as possible (PO: 467.05 ± 47.98 s) (*p* < 0.05, *d* = 0.38) or a solo exercise trial seeing only their own on‐screen avatar (SO: 470.07 ± 44.83 s) (*p* < 0.05, *d* = 0.41). Introducing a contemporary avatar with instruction to finish the trial as fast as possible did not improve finish time, as there was no difference to performing the same trial alone (*p* = 0.43, *d* = 0.14). The participants finished before their opponents in 21% of the PO trials, and 40% of the AO trials. The mean (± SD) chronological time measured pre‐exercise, during, and post‐exercise, and the RPE within the 4‐km trials with the different conditions (SO, PO, and AO) are presented in Figure 2. In the analysis of the measurement points (pre, during, post), the main effect for condition was not significant (*F*_2,64_ = 0.03, *p* = 0.97, *f* = 0.03) and neither was the interaction between conditions and measurement points (*F*_2.70,86.39_ = 0.46, *p* = 0.69, *f* = 0.12). The presence of the opponents, therefore, did not impact time perception (Figure 2a). In the analysis of the within‐trial measurement moments (500, 1500, and 2500 m), there was no main effect for condition (*F*_2,90 _= 0.06, *p* = 0.94, *f* = 0.03), nor was there a significant interaction effect (*F*_4, 90 _= 2.08, *p* = 0.09, *f* = 0.30) (Figure 2b). The trajectory of the RPE did not differ between the conditions (*F*_8, 488 _= 0.41, *p* = 0.92, *f* = 0.08) (Figure 2c).

## 4 DISCUSSION

The main finding from this study was that time distortion occurs during uncapped self‐paced exercise, compared to resting state, resulting in the perception of time apparently slowing down, that is, appears to run slower than the running, chronological clock. This effect was independent of the RPE, which is a novel finding. The current study is the first to use a standardized 30‐s test undertaken before, during and after all exercise bouts in competitive‐type situations. At rest, the estimate of time resulted in the perception that time moved fast (i.e. the chronological clock reaches 30‐s before the individual indicates/perceives 30‐s has elapsed, giving the impression that the chronological clock is moving [too] fast). This contrasts to the effects during exercise where it seems likely that the stimulus of physical activity creates a heightened associative state of impulse awareness and causes a perceived slowing of time (i.e. the chronological clock reaches 30 s after the individual indicates/perceives 30 s has elapsed, giving the impression that the chronological clock is moving [too] slow), consistent with the SBT model of time perception (Edwards & McCormick, 2017). This is also consistent with the Einstein's working example on the theory of relativity (Buhusi & Meck, 2009), where sitting on a hot stove meant that 1 min could seem like an hour (i.e. the chronological clock reaches 1 min too slow and slower than the individual estimates).

The results of this study using competitive exercise bouts also confirm previous observations of perceived slowing of time in capped (fixed RPE/physical exertion) trials (Cochrane et al., 2015; Edwards & McCormick, 2017). This is an important observation for application to practical exercise performance and optimizing strategies for winning races. In terms of perceived exertion, although the RPE increased from start to finish of each exercise trial, there was no association with changes in the time perception tests that were also undertaken at three standardized time points during the continuous 4‐km cycling trials. Therefore, the phenomenon that time appears to slow during exercise requires further investigation in terms of intensity and duration of activity. The slowing of perceived time during exercise persisted across exercise trial conditions (SO, PO, and AO), therefore suggesting that the level of engagement with a virtual opponent avatar might not provide a sufficient distraction to counteract the heightened associative state resulting from physical exercise. This is the first study to conclusively demonstrate that the perception of time slows down during exercise and is unaffected by the presence of fellow competitors and perceived exertion using a standardized, controlled experimental design.

While time perception is undoubtedly important to performance of sport, experimentally, it remains largely unexplored. Therefore, to accurately assess its impact on single and multiple athlete competition, we undertook extensive pre‐testing of experimental time perception protocols to extend work in this topic. To fully quantify the effects of exercise on time perception, a standardized approach to test a fixed duration estimate is required (Edwards & McCormick, 2017). To this end, numerous durations of fixed‐term prospective time estimates were trialed, noting that short duration estimates (e.g., <2–3 s) tend to rely on arousal and sensory processes, whereas longer duration estimates reflect cognitive and attentional mechanisms (Cochrane et al., 2015). Our testing demonstrated that 30‐s durations were not less accurate than 60‐s estimates.

Our observation that the perception of time was significantly slowed during exercise supports earlier work (Edwards & McCormick, 2017; Hanson & Lee, 2020). The effect of exercise on time perception demonstrated in this study is similar to situations of perceived threat often used in psychological research of time period estimation (Bar‐Haim et al., 2010). It has previously been shown that during dangerous incidents events appear to pass in slow motion as if time has slowed down (Eagleman et al., 2005) and in sports terms, this likely means that the subjective perception of time elapsed decreases (shrinks) due to greater than usual sensory awareness of physical sensations of discomfort that are not apparent at rest. Therefore, experiences or sensations (impulses) are densely packed into a shorter period than is objectively true compared to a resting condition due to augmented physical arousal and awareness of the physical situation. These observations could have important implications for how exercise training is designed to maximize enjoyment, engagement and thus healthy physical activity practices in a range of activities. However, in contrast to earlier studies, we did not find differences in RPE across conditions, suggesting exercise intensity was not a factor (Edwards & McCormick, 2017; Hanson & Lee, 2020). This is perhaps explained as our trials are the first to be conducted in truly self‐paced “all‐out” exercise and this contrasts with previous experiments where trials were performed at fixed effort levels corresponding to particular definitions of the Borg's 6–20 RPE scale (Morrone et al., 2005). That work suggested that higher intensity exercise leads to the greatest time distortion, which appears logical as this is where signaling of pain and discomfort from muscles to the brain are most apparent, leading to a more associative (i.e. aware and in‐the‐moment) state (Edwards & Polman, 2013). However, further work is required to support the argument of exercise intensity being a factor in time distortion rather than exercise per se. In our experiment, perceived time in all our exercise conditions and at all time points during exercise indicated the same effect indicating that perhaps it is exercise per se that significantly distorts time perception.

It has been postulated that distraction could be a useful technique of diverting attention from time perception and thus potentially divert attention away from painful physical sensations during exercise (Behm & Carter, 2020). Competitive opponents have been shown to affect athletes’ decision making, occupy focus, and potentially distract attention (Borg, 1962; Konings et al., 2020; Smits et al., 2014). In our experiment, we included exercise conditions without opponents (SO) or with opponents that were either passive contemporaries with no fixed instruction for our participants to engage with them (PO), and also as fellow competitors they should try to beat (AO). The OA trial was completed the fastest out of the three all conditions (*p* < 0.05), indicating that the intended active engagement with the opponent was achieved. However, interestingly this did not change participant ability to perform the time perception test at any interval. Accumulative RPE was also similar between each exercise trial and it seems likely that the main discerning factor influencing time perception is exercise per se rather than the presence of an opponent or the intensity. However, the fact that introducing opponents to the cycling trials did not counteract the slower of perceived time requires further discussion. The opponents used in this experiment were avatars using a process consistent with our previous experiments (Konings et al., 2016; Menting et al., 2019) and elicited similar physiological and psychological responses. Yet, counteracting the associative state induced by physical activity might require further variation in the perceived importance of the activity, the competitive environment, or the opposition itself (Hettinga et al., 2017). Previous studies have attempted to increase the importance of the activity and the engagement with the opponent by introducing a leader board (Konings et al., 2016), deceiving the participant to believe their opponent is another study volunteer (Konings & Hettinga, 2018), and restricting the participants to a maximum of one overtaking action (Konings et al., 2020). Alternatively, it should be noted as a limitation that the participants in the current study did not have experience with cycling tasks and although *n* = 33 is a strong sample size, the applicability of the results should be limited to the cohort demographics, pending wider sampling. Previous studies demonstrated that although novice cyclists are primarily concerned with finishing the task at hand, experienced cyclists are more focused on task performance (Boya et al., 2017). It could therefore be possible that the dependency upon the opponents, and the hypothesized distraction from the associative focus, would have been more pronounced in a sample of experienced cyclists.

This study is the first to experimentally demonstrate that time and relativity can be demonstrated through the medium of exercise with a standardized protocol, showing that time is distorted. This effect appears independent of the further presence of competitive or passive opponents; although the current study provides novel and impactful insights, more work has to be done to further unravel the role of external stimuli, exercise intensity, and duration on the perception of time during exercise. All of these factors affect timing, pacing, and the successful completion of optimal outcomes across physical activities.

## 5 CONCLUSIONS

In summary, this study has demonstrated that robust evaluation of time perception in response to exercise is differentially distorted compared to non‐exercise, resting evaluations. The presence of (virtual) opponents did not ameliorate some of the exercise‐induced slowing of perceived time (i.e., negative valence) by acting as a distraction of attentional focus in competitive situations, nor did it compound the distortion. This is the first study to have experimentally evaluated time perception using a standardized measurement test across truly self‐paced maximal exercise akin to real performance conditions and supports Einstein's example that time runs slower in certain situations, which in this case is sport. In terms of practical applications, accurate goal setting, planning, and in‐race awareness of chronological pace and time reinforced by external stimuli have recently been shown to be highly relevant to high level, world records. Therefore, further external reinforcement of accurate timing/pacing using novel techniques such as guided light systems like Wavelight could be helpful to aid athletes and coaches in achieving optimal outcomes. The impact of techniques to minimize time distortion and improve pacing are likely to be meaningful to both training and race performances.

## AUTHOR CONTRIBUTIONS

**Andrew Mark Edwards**: Conceptualization; investigation; writing—original draft; methodology; writing—review and editing; validation; formal analysis. **Stein Gerrit Paul Menting**: Conceptualization; investigation; writing—original draft; methodology; validation; writing—review and editing; formal analysis. **Marije Titia Elferink‐Gemser**: Conceptualization; investigation; methodology; writing—original draft; writing—review and editing. **Florentina Johanna Hettinga**: Conceptualization; investigation; writing—original draft; methodology; validation; writing—review and editing. All authors critically revised the work. All authors read and approved the final manuscript.

The authors would like to acknowledge the efforts of Mohammed Khudair in the process of collecting data for this experiment. The study conception and design were done in full collaboration with all authors. The authors declare that the results of the study are presented clearly, honestly, and without fabrication, falsification, or inappropriate data manipulation. Authors received no specific funding for this work.

## CONFLICT OF INTEREST STATEMENT

The authors declare no conflicts of interest.

## FUNDING INFORMATION

We can confirm no funding was received for this work.

### PEER REVIEW

The peer review history for this article is available at https://publons.com/publon/10.1002/brb3.3471.

## Data Availability

The data that support the findings of this study are available from the corresponding author upon reasonable request.
